# Revision of the Vietnamese millipede genus *Annamina* Attems, 1937, with descriptions of three new species (Diplopoda, Polydesmida, Paradoxosomatidae)

**DOI:** 10.3897/zookeys.669.12561

**Published:** 2017-04-20

**Authors:** Sergei I. Golovatch, Jean-Jacques Geoffroy, Nesrine Akkari

**Affiliations:** 1 Institute for Problems of Ecology and Evolution, Russian Academy of Sciences, Leninsky prospekt 33, Moscow 119071 Russia; 2 Muséum national d’Histoire naturelle, DSE, Site de Brunoy, 4 Avenue du Petit Château F-91800 Brunoy, France; 3 Naturhistorisches Museum Wien, Burgring 7, A-1010 Wien, Austria

**Keywords:** Diplopoda, Paradoxosomatidae, *Annamina*, taxonomy, new species, Vietnam

## Abstract

The hitherto monotypic diplopod genus *Annamina* contains now four species, including the revised type-species *A. xanthoptera* Attems, 1937, as well as *A. attemsi* **sp. n.**, *A. irinae* **sp. n.** and *A. mikhaljovae* **sp. n.**, all from central or southern Vietnam. The genus is rediagnosed and a key to its constituent species given.

## Introduction

The monotypic millipede genus *Annamina* Attems, 1937, and its type species *A. xanthoptera* Attems, 1937, were described twice nearly simultaneously from Vietnam, first in a global review of the family Paradoxosomatidae (= Strongylosomatidae) (Attems 1937) and the second time in a large paper on the Myriapoda of Indochina (Attems 1938). Although the designation of *Annamina xanthoptera* as a new genus and species appeared in 1938, the actual publication date was 1937.

The genus *Annamina* belongs to the tribe Sulciferini Attems, 1898 and differs from the other, mostly Asian genera by a remarkably short flagelliform solenomere and an even shorter, chitinous, tooth-shaped solenophore devoid of membranous elements (Jeekel 1968). *Annamina xanthoptera*, the only hitherto known species of the genus, was described from near Danang, central Vietnam (Attems 1937, 1938) and has never been recorded since.

Recently, three new *Annamina* species were discovered in southern and central Vietnam. One of these, *A. attemsi* sp. n., was incidentally found by NA when documenting the type material of *A. xanthoptera* in the Naturhistorisches Museum Wien (NHMW). In this work, we redescribe *A. xanthoptera*, based on the type material, and select a lectotype to stabilize the species’ taxonomy. We also describe three new species: *A. attemsi* sp. n., *A. irinae* sp. n. and *A. mikhaljovae* sp. n., as well as refine the diagnosis of the genus and provide a key to all four species known to date.

## Material and methods

New material that contained two new species of *Annamina* was taken in 2015 and 2016 during field trips of I. I. Semenyuk (Moscow, Russia) to southern Vietnam in the framework of the research activities of the Joint Russia-Vietnam Tropical Centre. The types of both are deposited in the Zoological Museum of the Moscow State University, Russia (ZMUM). The ZMUM types are preserved in 75% ethanol. Pictures were taken with a Canon EOS 5D digital camera and stacked using Zerene Stacker software.

The NHMW types are preserved in 75% alcohol. Measurements and photographs were taken with a Nikon DS-F2.5 camera mounted on a Nikon SMZ25 stereo microscope, using NIS-Elements Microscope Imaging Software with an Extended Depth of Focus (EDF) patch. All images were processed with Adobe Photoshop CS6 and assembled in Adobe InDesign. For taking SEM micrographs, the gonopods of *A. xanthoptera* and *A. attemsi* sp. n. were dehydrated in 96% ethanol and acetone, then air dried, mounted on aluminium stubs, coated with platinum and examined using a JEOL JSM 6610 scanning electron microscope.

Several paralectotypes of *A. xanthoptera*, preserved in 70% alcohol, are housed in the Muséum national d’Histoire naturelle, Paris (MNHN). They were only measured and checked for their species identity.

## Results

### Taxonomy

#### Polydesmida Leach, 1815

##### Paradoxosomatidae Daday, 1889

###### Genus Annamina

Taxon classificationAnimaliaPolydesmidaParadoxosomatidae

Attems, 1937

####### Diagnosis.

Medium-sized (ca 2–3 cm long) Sulciferini with 20 body segments, distinct, thin and mostly subhorizontal paraterga, evident transverse sulci on metaterga 5–17(18), and a very high, tongue-shaped, setose, subtruncate lobe between ♂ coxae 4.

Gonopod mostly stout, prefemoral (= densely setose) region small, much shorter than femorite, the latter usually with evident, sometimes hyline, mesal and/or ventral lobes, clearly set off by sulci from both pre- and postfemoral parts; seminal groove mostly dorsal, not mesal, running onto a short flagelliform solenomere on mesal face near distal (= postfemoral) sulcus; acropodite consisting of a prominent central spine sometimes flanked by a mesal and/or a lateral process/outgrowth and carrying parabasally or near midway an inconspicuous, short, dentiform, ventral solenophore devoid of membranous elements and subtending the distal part of solenomere.

####### Type species.

*Annamina xanthoptera* Attems, 1937

####### Other species included.

*A. attemsi* sp. n., *A. irinae* sp. n. and *A. mikhaljovae* sp. n.

###### Annamina xanthoptera

Taxon classificationAnimaliaPolydesmidaParadoxosomatidae

Attems, 1937

Figs 1 , 2 , 3 , 4

####### Type material.

NHMW: Lectotype ♂, NHMW 8936, designated herein, Tourane (= Danang), Lien Chieu, Dawydoff C. leg., 09.1931, Dawydoff/Attems 1936 don., Attems det. Paralectotypes: 4 ♂♂, 6 ♀♀, 3 heads, 3 posterior sections, several midbody sections, NHMW 8937, two slide preparations, NHMW3477, same data as lectotype. MNHN JA 108: 2 ♂♂, 3 ♀♀, Touranne (C. Annam), 18.IX.31 Lien-Chiên.

Lectotype designation was necessary so that the species is based on a complete male that fully matches the original description of *A. xanthoptera* by Attems (1937). Gonopods were removed and newly examined using one of the NHMW paralectotypes.

####### Diagnosis.

Differs from other members of the genus primarily by showing both the median lobe and the lateral process of the gonopod telopodite strongly microdenticulate-serrate. See also Key below.

####### Description.

Measurements (mm): Males (both NHMW and MNHN): length 24.9–29, width of midbody prozonae 1.6–1.9, width of midbody metazonae 2.35–2.6. Females (both NHMW and MNHN): length 28–31, width of midbody pro- and metazonae 1.8–2.1 and 2.5–3.2, respectively.

**Figure 1.**
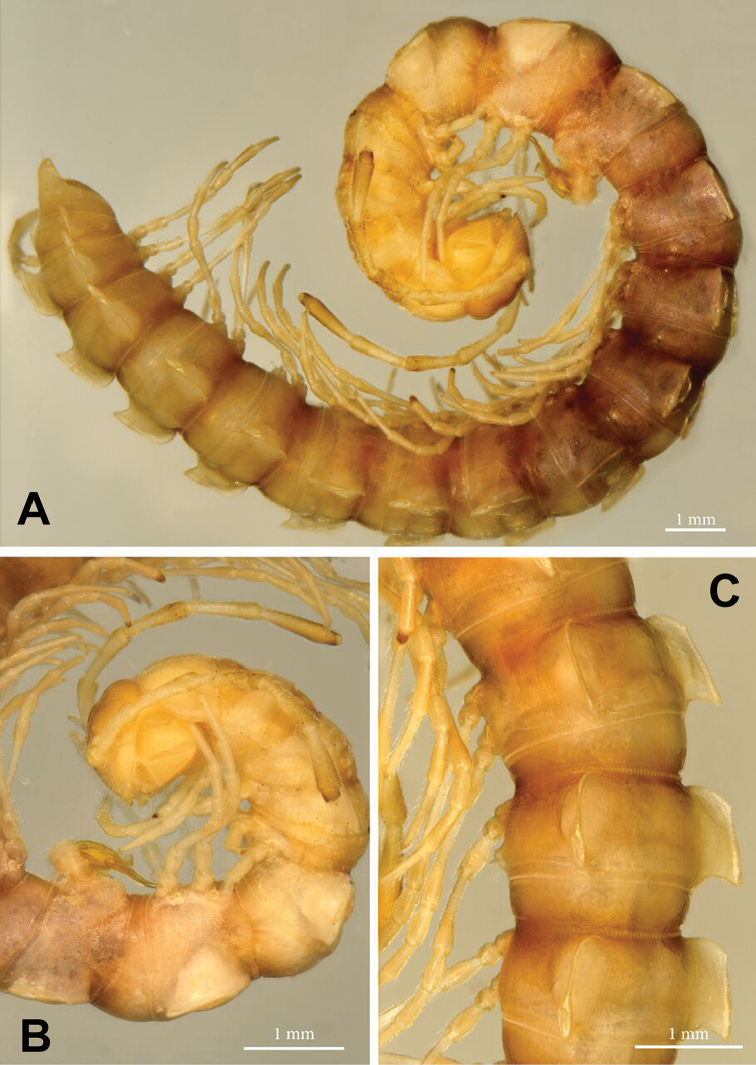
*Annamina xanthoptera* Attems, 1937, ♂ paralectotype (NHMW). **A** habitus, lateral view **B** anterior part of body, lateral view **C** midbody segments, dorsolateral view.

General coloration after many years of preservation in alcohol apparently somewhat faded, rather uniformly light to castaneous brown, without a distinct pattern, sides lighter; telson, legs and ventral parts light brown to yellowish (Fig. 1). Clypeolabral region setose, setae becoming scattered between antennae; vertigial region with 2+2 setae; epicranial suture thin, superficial.

**Figure 2.**
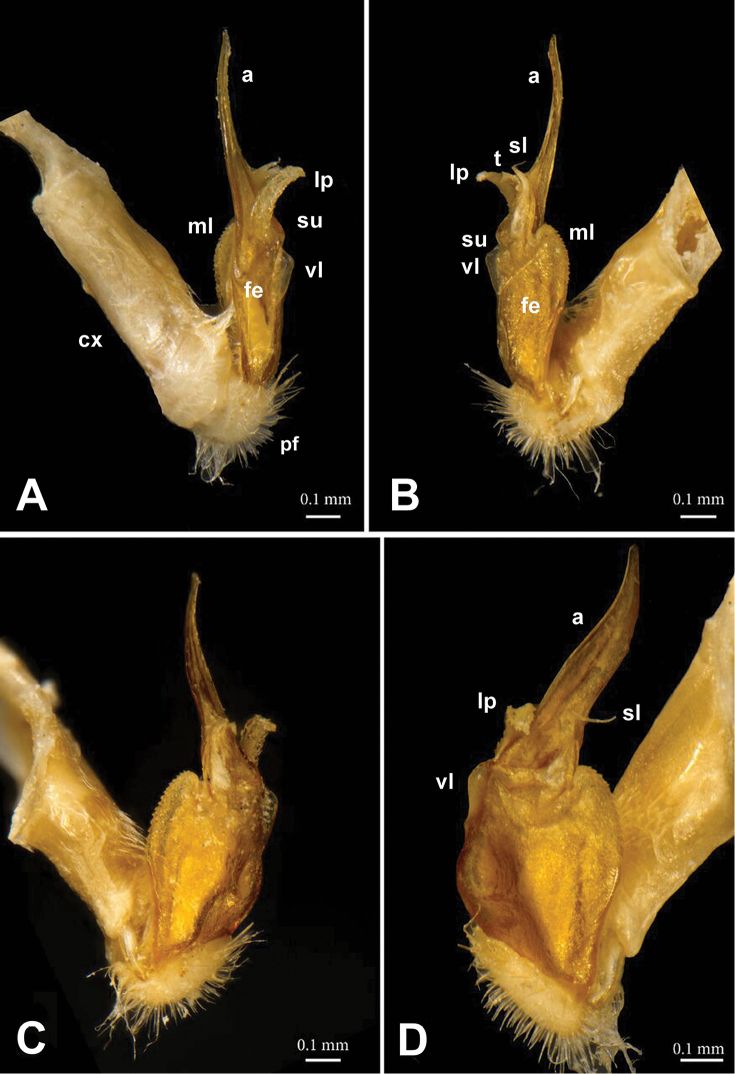
*Annamina xanthoptera* Attems, 1937, ♂ paralectotype (NHMW), right (**A, B, D**) and left (**C**) gonopods, **A** lateral **B** mesal **C** ventromesal and **D** subventral views, respectively (cx = coxite; fe = femorite; pf = prefemoral part; vl = ventral lobe; ml = mesal lobe; su = postfemoral sulcus; lp = lateral process; a = apical process; sl = solenomere; t = solenophore tooth).

Antennae long, slender and moderately clavate, slightly extending back behind segment 3 (♂) (Fig. 1A, B) or 2 (♀) when stretched dorsally; in length, antennomere 2 = 3 = 4 = 5 = 6 > 1 = 7 (Fig. 1A, B). In width, collum = segment 3 = 4 < 2 < head = 5–16 (♂); thereafter body gradually tapering towards telson on segments 17–19. Tegument generally smooth, prozonae finely shagreened, rear halves of metaterga mostly striolate; surface below paraterga microgranulate. Collum regularly rounded laterally; dorsum strongly and regularly convex, but paraterga directed ventrolaterad. Postcollum paraterga well-developed, mostly set high (at about 1/5 metazonital height measured from dorsum), subhorizontal; paraterga 2 lower than others, drawn both forward and caudad into rounded lobes, with a distinct lateral tooth in fore 1/4; following paraterga broadly and regularly rounded anterolaterally, likewise with a small, but evident tooth in fore 1/4; caudal corner subrectangular until segment 5, increasingly dentiform and well drawn caudad, but evidently projecting behind caudal tergal margin only in segments 17–19, nearly always rounded, spiniform and almost pointed only in segment 19; calluses narrow, demarcated by a complete, distinct, deep sulcus only dorsally and by a faint and somewhat incomplete one ventrally, the latter sulcus reaching only until fore lateral tooth; poriferous calluses only a little thicker than poreless ones (Fig, 1). Ozopores lateral, placed inside an elongated ovoid groove located just behind a vague tubercle at about rear 1/4 callus. Transverse metatergal sulci thin, shallow, faintly sinuate medially and beaded at bottom, nearly reaching bases of paraterga, present on metaterga 5–18 (Fig. 1). Stricture dividing pro- and metazonae thin and deep, ribbed at bottom down to paraterga. Axial line very faint, traceable in places on metaterga. Pleurosternal carina a small ventral lobule on segment 2, thereafter very faint, subtransverse, granulated ridges traceable caudally until segment 7 (♂). Epiproct (Fig. 1A) long, clearly flattened dorsoventrally, conical, emarginate at apex, subapical lateral papillae small. Hypoproct subtriangular, with a rounded apex, caudal 1+1 setae well-separated, not borne on knobs (as in Fig. 11A).

Sterna flat, sparsely setose, cross-impressions faint, without modifications other than a prominent, very high, narrow, triangular, truncate lobe between ♂ coxae 4 (as in Fig. 11B). Legs long, ca 2 times as long as midbody height, very slender in both sexes, with neither adenostyles nor ventral brushes; in length, femora > prefemora > tarsi > coxae = postfemora = tibiae (Fig. 1).

**Figure 3.**
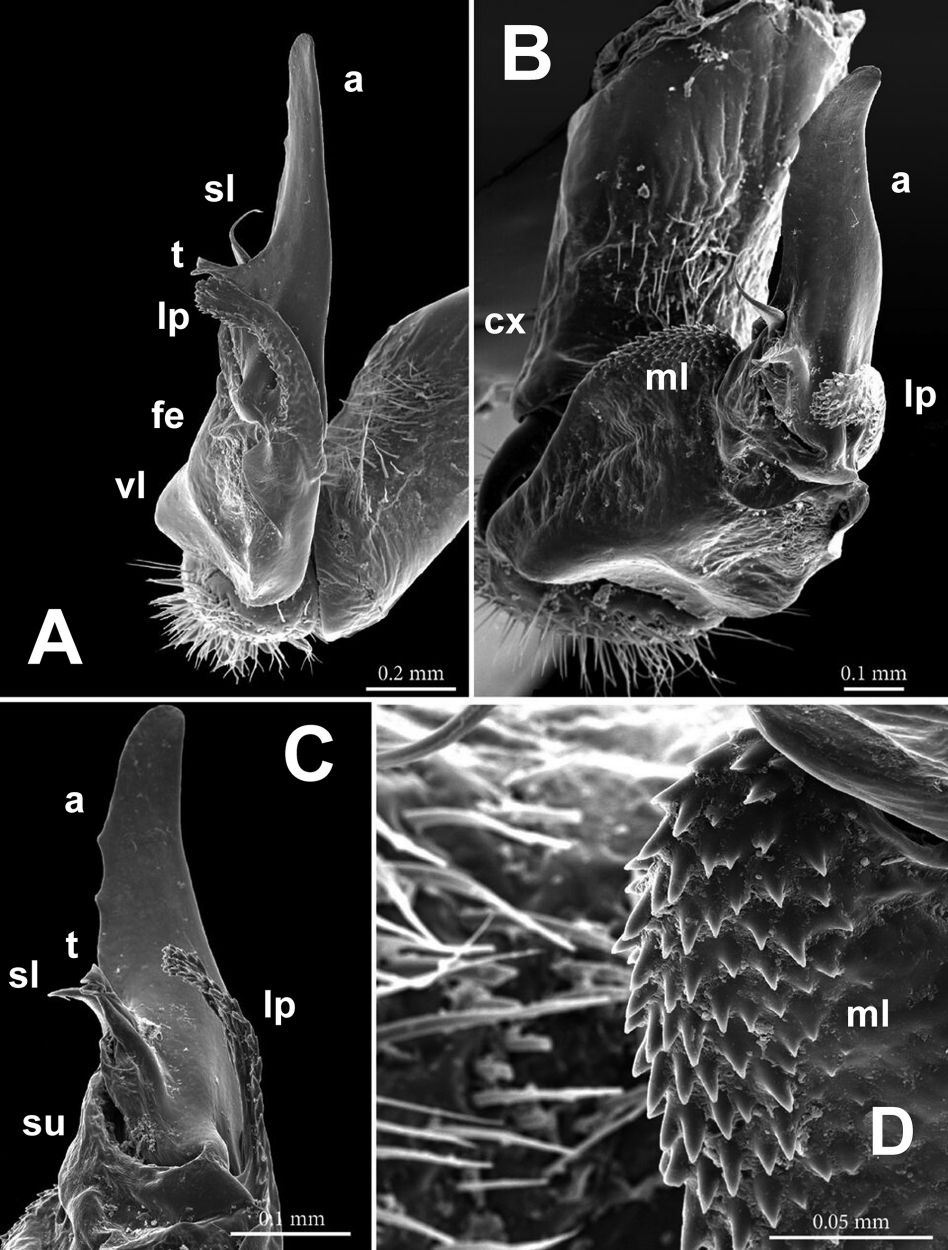
*Annamina xanthoptera* Attems, 1937, ♂ paralectotype (NHMW), SEM micrographs of entire left gonopod (**A, B**), ventrolateral and subventral views, respectively, and of its distal parts (**C, D**), subventrolateral views (cx = coxite; fe = femorite, vl = ventral lobe; ml = mesal lobe; su = postfemoral sulcus; lp = lateral process; a = apical process; sl = solenomere; t = solenophore tooth).

**Figure 4.**
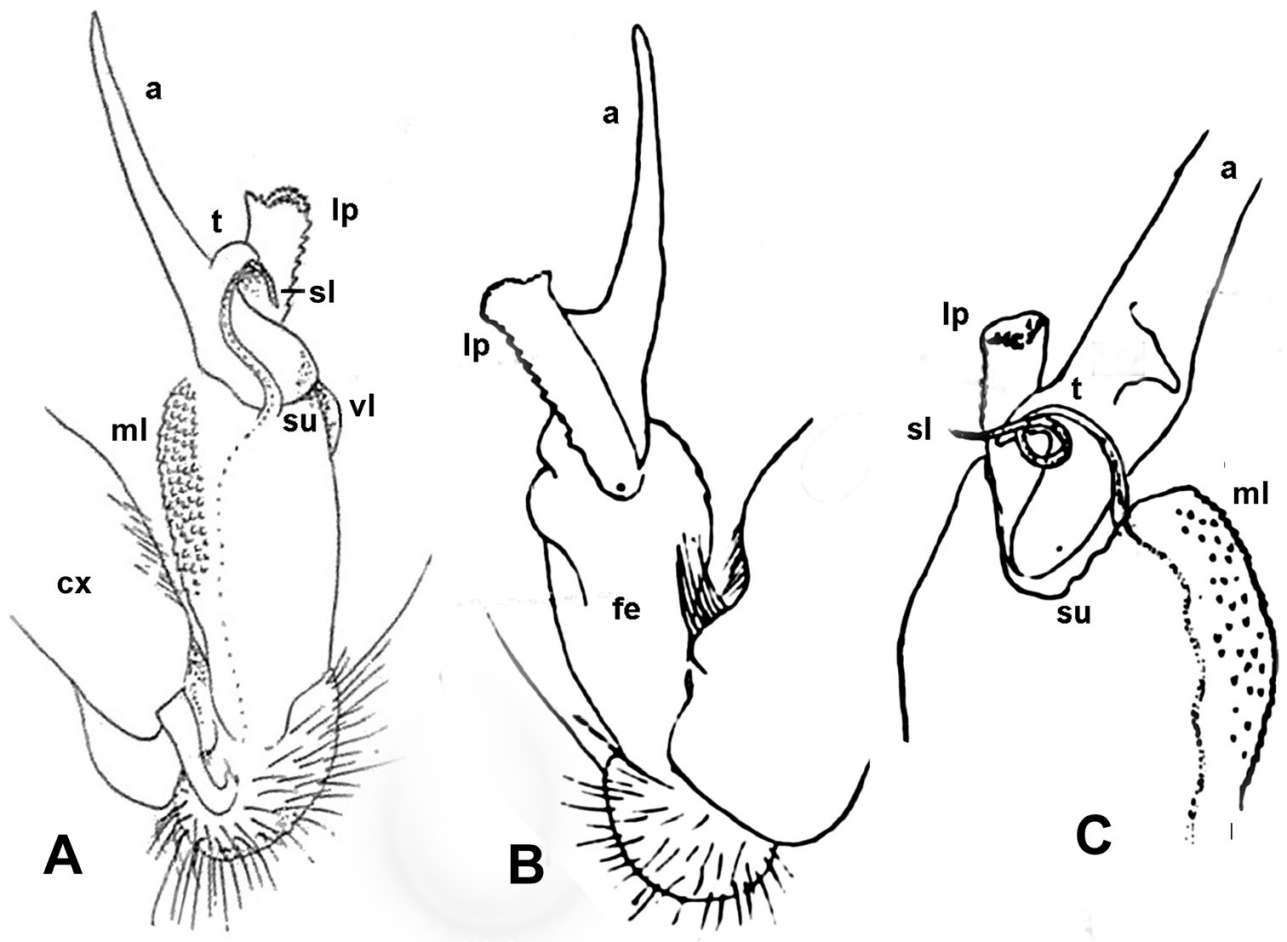
*Annamina xanthoptera* Attems, 1937, ♂ paralectotype (NHMW), entire left (**A, B**) gonopod and distal part of right one (**C**), mesal, lateral and subventral views, respectively (cx = coxite; fe = femorite, vl = ventral lobe; ml = mesal lobe; su = postfemoral sulcus; lp = lateral process; a = apical process; sl = solenomere; t = solenophore tooth). After Attems (1937) (**A**) and Attems (1938) (**B, C**).

Gonopods (Figs 2–4) complex, telopodites stout. Coxite (**cx**) considerably shorter than telopodite, subcylindrical, densely setose distoventrally. Prefemoral (= densely setose) part (**pf**) short, set off from femorite (**fe**) by an oblique sulcus. Femorite (**fe**) voluminous, clearly flattened dorsoventrally, showing a prominent, spiculate-microdenticulate, mesal lobe (**ml**) and a smaller, rounded, hyaline, ventral lobe (**vl**); seminal groove running laterad along dorsal part of **fe**, distally detached near a subtransverse postfemoral sulcus (**su**) into a conspicuously short, flagelliform, coiled solenomere (**sl**). On ventral side, base of **sl** subtended by a small tooth (**t**) (= solenophore) devoid of membranous elements, **t** lying ventrally near base of a long, narrow, blade-shaped, apical process (**a**); the latter slightly curved laterad, with a rounded tip, much longer than a conspicuously serrate, slender, finger-shaped, lateral process (**lp**).

####### Comments.

Attems (1937, 1938) failed to indicate the number of specimens in the type series of *A. xanthoptera* while the only measurements he gave in the descriptions (width of pro- and metazona 1.8 and 2.5 mm, respectively) may have misleadingly been taken as concerning a single specimen. However, the type series is quite large and presently divided between the MNHN and NHMW collections. Moreover, the NHMW type material actually houses two different species of *Annamina*, most of which truly represents *A. xanthoptera*. The minor admixture, however, is described below as still another new species, the types being deposited in the NHMW.

A complete catalogue of references to *A. xanthoptera* is available in Nguyen and Sierwald (2013).

###### Annamina attemsi sp. n.

Taxon classificationAnimaliaPolydesmidaParadoxosomatidae

http://zoobank.org/25C4DF37-7438-49B4-8764-EF365CA11F9F

Figs 5 , 6 , 7

####### Type material.

Holotype ♂, NHMW 8934, Tourane (= Danang), Lien Chieu, Dawydoff C. leg., 09.1931, Dawydoff/Attems 1936 don. Paratype: 1 ♂, NHMW 8935, with one gonopod dissected, same data as holotype.

####### Diagnosis.

Differs from other species of the genus primarily by an unusually slender telopodite of the gonopod which is only supplied with an apical process. See also Key below.

####### Name.

Honours the famous Austrian myriapodologist Carl Attems (1868–1952), one of the most prominent taxonomists of Diplopoda of his time.

####### Description.

Measurements (mm): Body length ca 18.7 (holotype) or 21.6 (♂ paratype), width of midbody prozonae 1.2 (holotype) or 1.3 (♂ paratype), width of midbody metazonae 1.8 (holotype) or 2.0 (♂ paratype).

**Figure 5.**
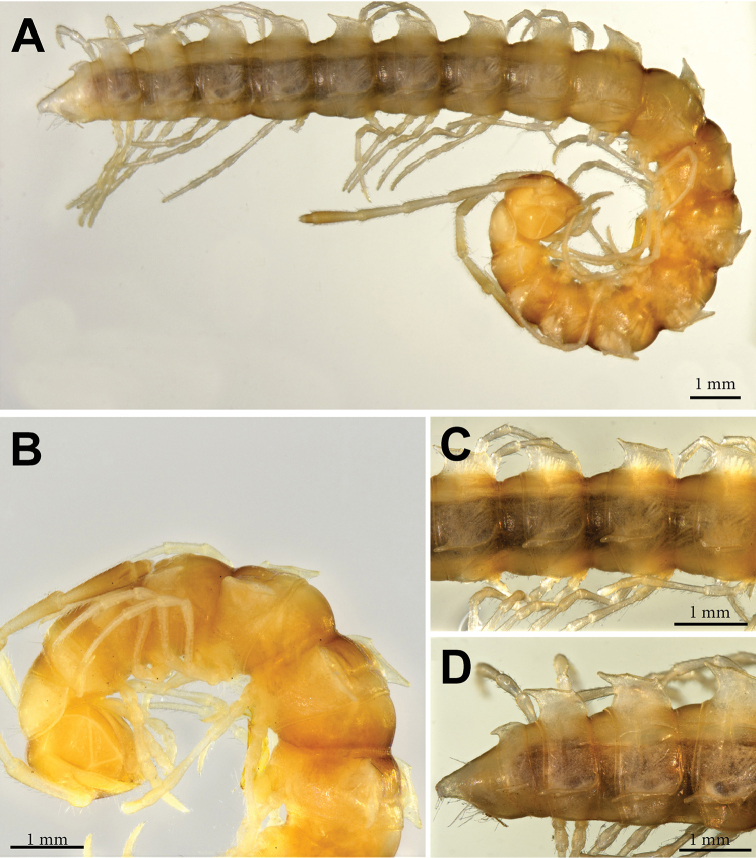
*Annamina attemsi* sp. n., ♂ paratype (NHMW). **A** habitus, lateral view **B** anterior part of body, lateral view **C** midbody segments, dorsolateral view **D** caudal part of body, dorsolateral view.

General coloration after many years of preservation in alcohol light, almost whitish to yellowish brown, sides, telson, legs and ventral parts pale whitish (Fig. 5). Clypeolabral region setose, setae becoming scattered between antennae (ca 3 pairs), vertigial region with 2+2 setae; epicranial suture thin, superficial.

**Figure 6.**
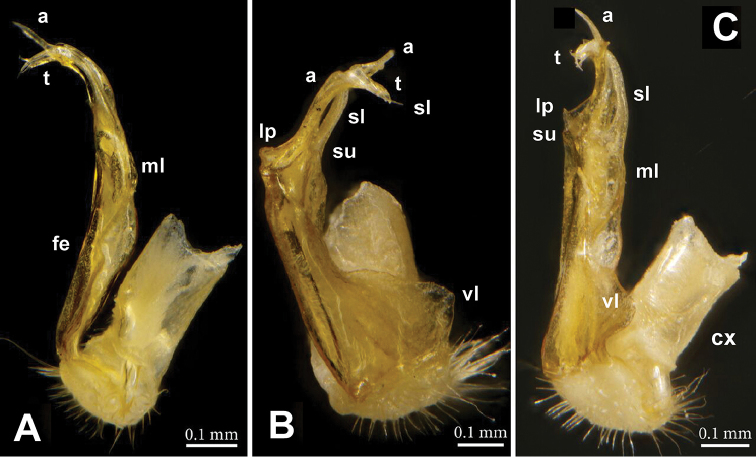
*Annamina attemsi* sp. n., ♂ paratype (NHMW), right gonopod, **A** mesal **B** subventral and **C** submesal views, respectively (cx = coxite; fe = femorite, vl = ventral lobe; ml = mesal lobe; su = postfemoral sulcus; lp = lateral process; a = apical process; sl = solenomere; t = solenophore tooth).

**Figure 7.**
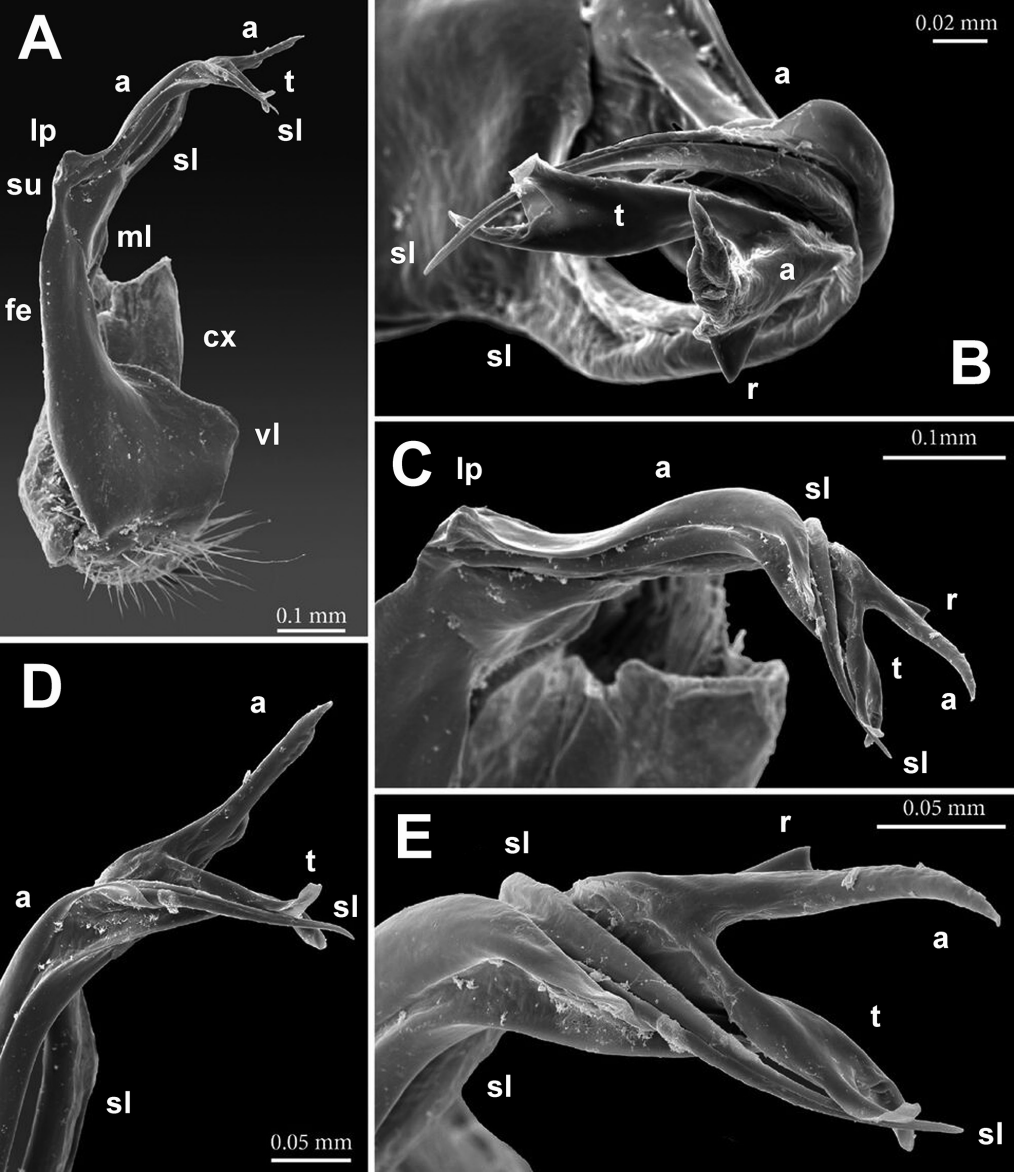
*Annamina attemsi* sp. n., ♂ paratype (NHMW), SEM micrographs. **A** entire right gonopod, ventral view **B–E** distal part of same gonopod **B** oral **C** subventral **D** ventral and **E** subventral views, respectively (cx = coxite; fe = femorite, vl = ventral lobe; ml = mesal lobe; r = subapical ridge; su = postfemoral sulcus; lp = lateral process; a = apical process; sl = solenomere; t = solenophore tooth).

All other characters (see Figs 5–7) as in *A. xanthoptera*, except as follows.

Caudal corner of paraterga dentiform and acute-angled starting with segment 4, drawn behind rear tergal margin starting with segment 11 (♂); a second lateral denticle on paraterga completely absent from segments 18 and 19. Transverse sulci fully developed on metaterga 5–17, deep, beaded at bottom and almost reaching the bases of paraterga, weaker on segment 18, absent from 19^th^.

Gonopods (Figs 6, 7) somewhat disjunct, especially intricate in structure; coxite (**cx**) moderately setose distoventrally; telopodite much more slender than in *A. xanthoptera*; femorite (**fe**) much narrower, distally especially so, showing an inconspicuous and less strongly granulated mesal lobe (**ml**), as well as a hypertrophied, hyaline, irregularly rounded, ventral lobe (**vl**) folded mesad; seminal groove running laterad along dorsal part of **fe**, passing distally onto a short flagelliform solenomere (**sl**); the latter subtended distally by a small, apically bilobulate, ventral tooth (**t**) (= solenophore) arising from about midway of a long, pointed, spiniform, apical process (**a**), this being equipped with a minute, subapical, hyaline ridge (**r**); lateral process (**lp**) rudimentary, lying a little distal to postfemoral sulcus (**su**), with a minute spike on top, placed just at base of **a**; no mesal process whatsoever.

###### Annamina irinae sp. n.

Taxon classificationAnimaliaPolydesmidaParadoxosomatidae

http://zoobank.org/44D470B6-EB4D-473F-8B15-E7626B4CB9F7

Figs 8 , 9

####### Type material.

Holotype ♂, ZMUM ρ3548, Vietnam, Gia Lai Prov., Kon Ka Kinh National Park, N 14°12'43.4", E 108°18'57.1", 930 a.s.l., humid leaved tropical forest in river valley, beaten from bush, V.2016, leg. I.I. Semenyuk. Paratypes: 2 ♂♂, ZMUM ρ3549, same locality and habitat; 1 ♂, ZMUM ρ3550, same locality artificial Pinus cf. kesia plantation, V.2016, leg. I.I. Semenyuk.

####### Name.

Honours Irina Semenyuk, the collector.

####### Diagnosis.

Differs from other species of the genus primarily by the presence of a small ventral lobe and a large mesal lobe on the gonopod femorite, coupled with a small, simple, mesal process, a long, spiniform, lateral process and a prominent, simple, unciform, acuminate, apical process in the postfemoral portion of the gonopod. See also Key below.

####### Description.

Measurements (mm): Body length of all types ca 24, width of midbody pro- and metazona 1.5 and 1.9, respectively.

**Figure 8.**
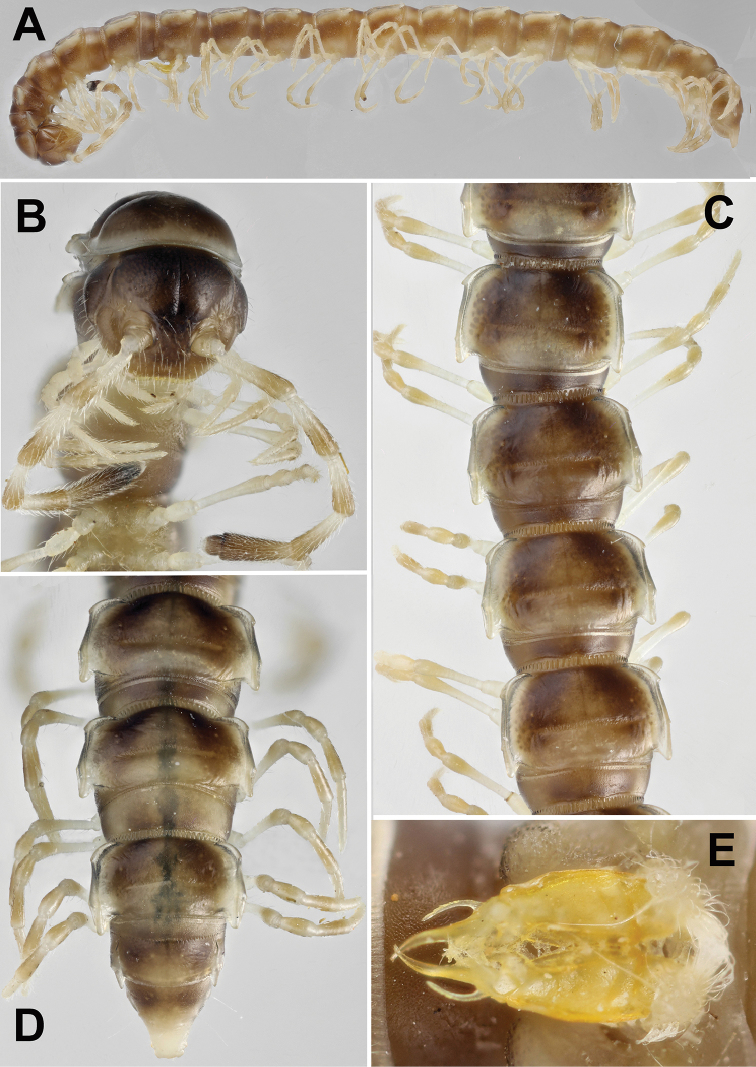
*Annamina irinae* sp. n., ♂ paratype (ZMUM). **A** habitus, lateral view **B** anterior part of body, anteroventral view **C** midbody segments, dorsal view **D** caudal part of body, dorsal view **E** gonopods, ventral view. Pictures taken not to scale.

General coloration in alcohol light brownish to brown, but with a characteristic pattern of a vague, lighter, subtriangular, central spot on each postcollum metatergum flanked on each side by marbled brown patches fused into a complete transverse band in anterior 1/3; sides mostly brown to light brown, lighter closer to coxae; strictures between pro- and metazonae, paraterga both dorsally and ventrally, telson, legs and venter pale yellowish to whitish (Fig. 8). Antennae nearly pallid, only distal parts of antennomeres 2–5 slightly infuscate, light brown; entire antennomeres 6 and 7 brown; tip of antennae contrasting pallid.

**Figure 9.**
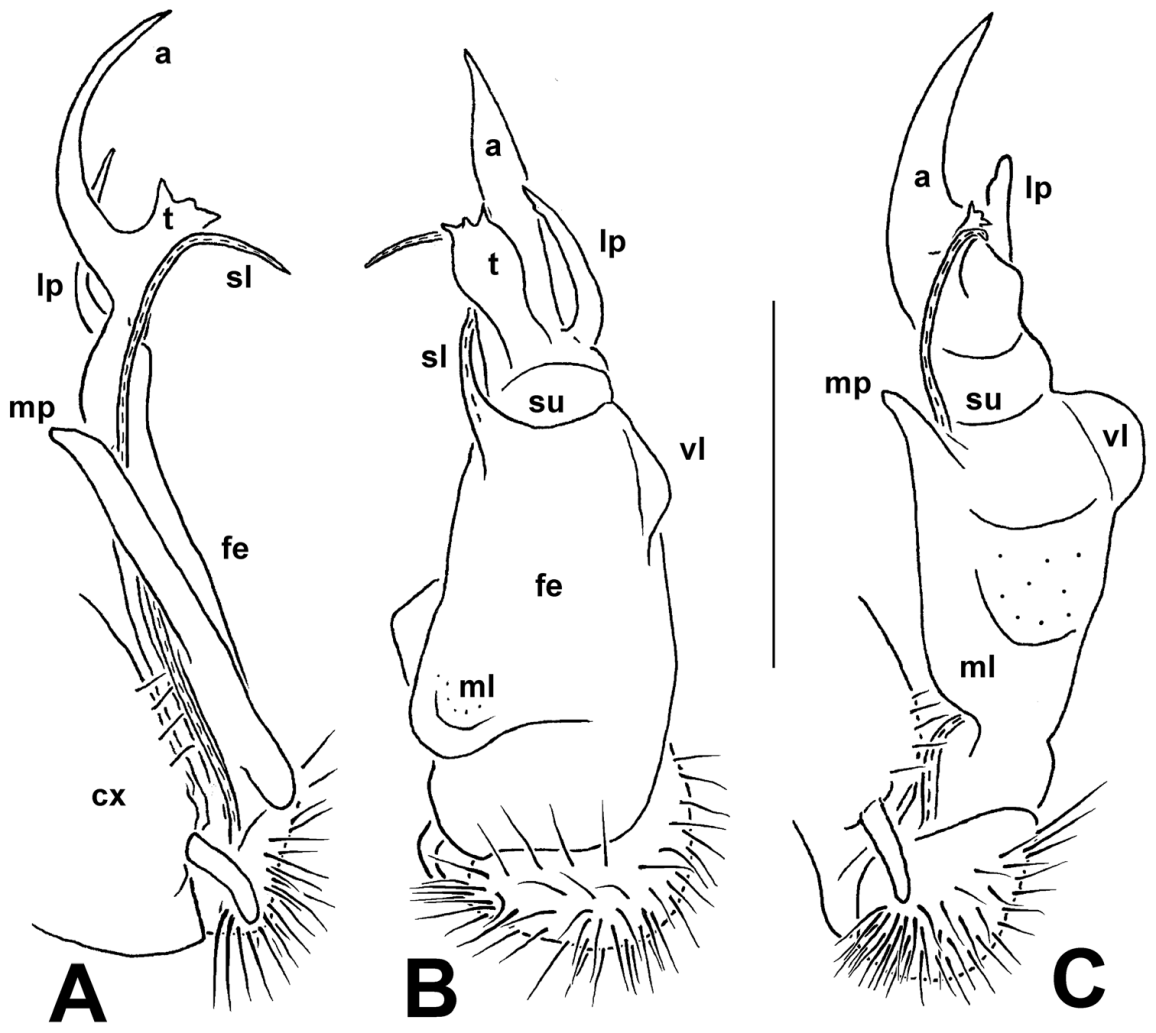
*Annamina irinae* sp. n., ♂ paratype (ZMUM), left gonopod, **A** dorsomesal, **B** ventral and **C** mesal views, respectively (cx = coxite; fe = femorite, vl = ventral lobe; ml = mesal lobe; su = postfemoral sulcus; lp = lateral process; mp = mesal process; a = apical process; sl = solenomere; t = solenophore tooth). Scale bar 0.5 mm.

All characters (see Figs 8, 9) as in *A. xanthoptera*, except as follows.

In width, segments 5–16 > head > 2 > collum = 3 = 4 (♂); body gradually tapering towards telson on segments 17–19 (Fig. 8B–D). Caudal corner of paraterga clearly drawn behind rear tergal margin on segments 16–19. Transverse sulci thin, slightly sinuate medially, finely beaded at bottom, fully developed on segments 5–17, weaker on segment 18, absent from 19^th^. Tergal setae ca 1/4 as long as metatergum, mostly abraded, often untraceable even as insertion points, pattern 2+2 in a transverse fore row. In length, midbody femora > tarsi > postfemora > coxae = prefemora = tibiae (Fig. 8B–D).

Gonopods (Figs 8E, 9) relatively complex; coxite (**cx**) moderately setose distoventrally; telopodite consisting of a short prefemoral (= densely setose) part set off from femorite (**fe**) by an oblique sulcus; femorite (**fe**) stout, distinctly flattened dorsoventrally, set off from acropodite by a subtransverse postfemoral sulcus (**su**), with a smaller, rounded, hyaline, ventral lobe (**vl**) and a much larger, parabasal, somewhat ear-shaped, mesal lobe (**ml**); seminal groove quickly moving laterad to mostly lie on dorsal side of **fe** before passing onto a short, free, flagelliform solenomere (**sl**) near **su**, with a small finger-shaped mesal process (**mp**) lying at base of **sl**; postfemoral part consisting of a large, spiniform, slightly curved, acuminate, apical process (**a**) flanked by a considerably shorter, likewise spiniform and slightly curved lateral process (**lp**) and a short, stout, ventral, trifid tooth (**t**) (= solenophore) subtending the basal half of **sl**.

###### Annamina mikhaljovae sp. n.

Taxon classificationAnimaliaPolydesmidaParadoxosomatidae

http://zoobank.org/C53AD3C4-4A6B-4F16-A8A0-BAC2ABB23F00

Figs 10 , 11

####### Type material.

Holotype ♂, ZMUM ρ3551, Vietnam, Kon Tum Prov., Kon Plong Distr., N14°43.450', E108°18.882', 1000–1260 m a.s.l., tropical forest, on log, V.2015, leg. I.I. Semenyuk.

####### Name.

Honours Elena Mikhaljova, a prominent specialist in the systematics of Asian Diplopoda.

####### Diagnosis.

Differs from other species of the genus primarily by the presence of a small ventral lobule and a large mesal lobe on the gonopod femorite, coupled with, much like in *A. irinae* sp. n., a small, simple, mesal process, a similarly short, but spiniform, clearly serrate lateral process and a prominent, lobe-shaped, apical process in the postfemoral portion of the gonopod. See also Key below.

####### Description.

**Figure 10.**
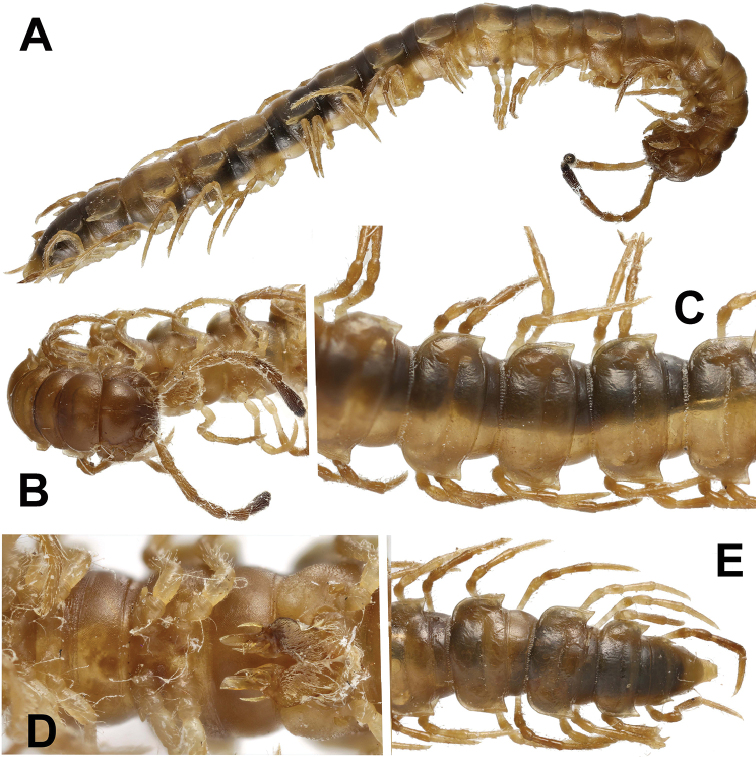
*Annamina mikhaljovae* sp. n., ♂ holotype (ZMUM). **A** habitus, lateral view **B** anterior part of body, ventral view **C** midbody segments, dorsal view **D** segments 5–7, ventral view **E** caudal part of body, dorsal view. Pictures taken not to scale.

Measurements (mm): Length ca 22 mm, width of midbody pro- and metazonae 1.8 and 2.7 mm, respectively. Coloration uniformly light brownish to yellow-brown, only antennomeres 6 and 7 contrasting dark brown; tegument largely thin and translucent (Fig. 10).

**Figure 11.**
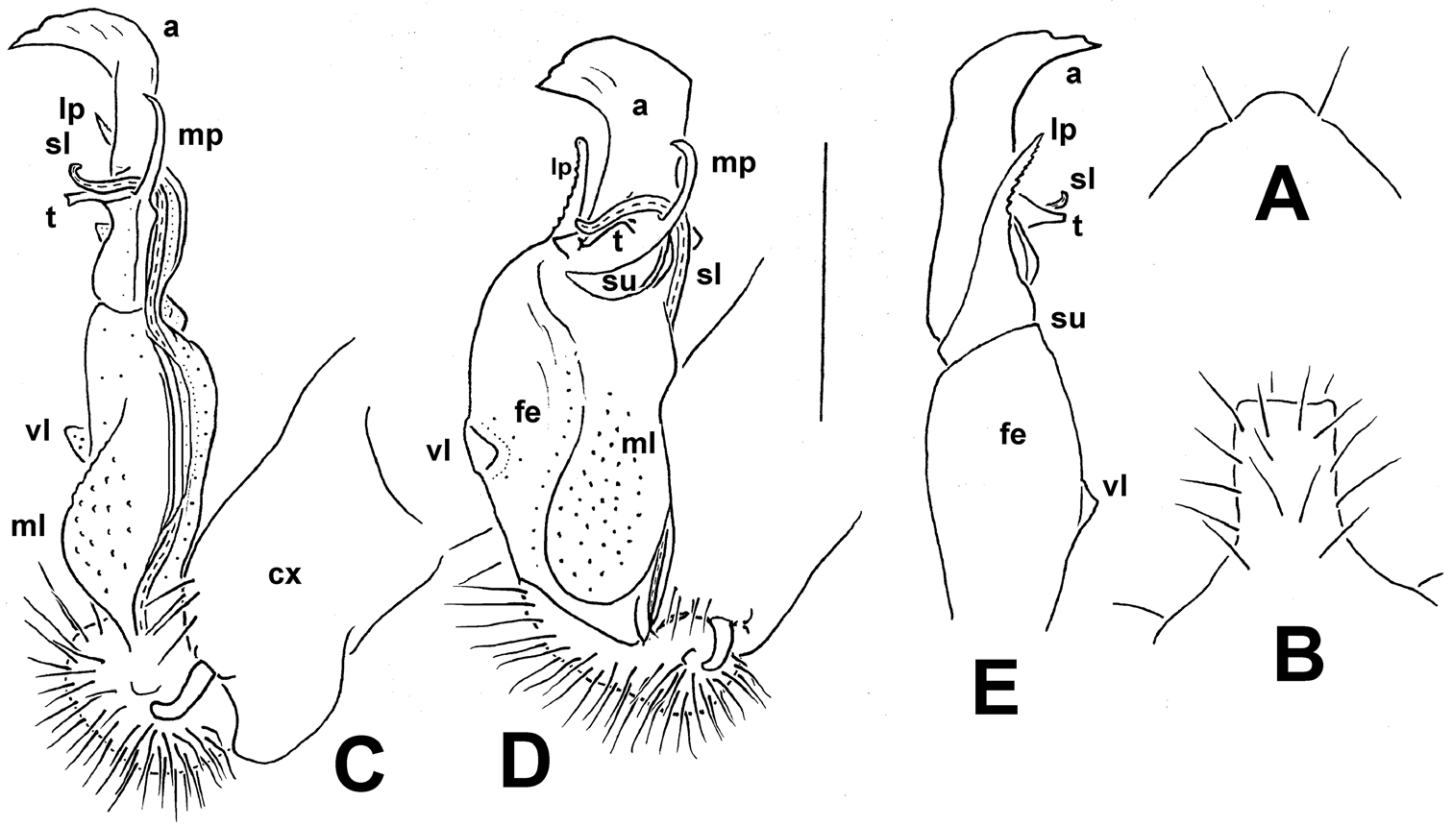
*Annamina mikhaljovae* sp. n., ♂ holotype (ZMUM). **A** hypoproct, ventral view **B** sternal lobe between coxae 4 **C–E** right gonopod, **A** mesal, **B** subventral and **C** lateral views, respectively (cx = coxite; fe = femorite, vl = ventral lobe; ml = mesal lobe; su = postfemoral sulcus; lp = lateral process; mp = mesal process; a = apical process; sl = solenomere; t = solenophore tooth). Scale bar 0.5 mm.

All characters (see Figs 10, 11) as in *A. xanthoptera*, except as follows.

In width, collum = 3 = 4 < segment 2 < head < 5–16 (♂); thereafter body gradually tapering towards telson. Caudolateral corner of paraterga subrectangular until segment 8, thereafter increasingly well drawn caudad, but always remaining narrowly rounded, clearly projecting behind rear tergal margin only on segments 17–19 (Fig. 10).

Gonopods (Figs 10D, 11) much as in *A. irinae* sp. n.; femorite (**fe**) with a distinct, papillate, parabasal, mesal lobe (**ml**), a small, subtriangular, midway, hyaline, ventral lobe (**vl**); postfemoral part lying beyond a distinct sulcus (**su**) with a short flagelliform solenomere (**sl**), a similarly short, slender, slightly curved, mesal process (**mp**), a short tooth (**t**) (= solenophore) subtending the basal portion of **sl**, a prominent, membranous, distally faintly serrate, lobe-shaped, laterad curved, acuminate, apical process (**a**), and a shorter, spiniform, slearly serrate, lateral process (**lp**).

## Discussion

Even with such a considerable increase in species diversity as described above, *Annamina* remains a well-defined and quite homogeneous group within the paradoxosomatid tribe Sulciferini. The genus is remarkably uniform in most of the characters of its constituent species, both somatic and gonopodal, as well as in distribution which is confined to south-central and central Vietnam. *Annamina* is unique amongst the contribal genera in the high, well-developed, laterally mostly monodentate and caudally largely triangular (but never pointed) paraterga; the strongly ribbed strictures between pro- and metazonae; the microgranulate sides of metazonae; the inconspicuous, nearly missing pleurosternal carinae; the uniformly roundly subtriangular hypoproct; the unusually high, subtriangular, setose and apically truncate sternal lobe between ♂ coxae 4; the absence both of adenostyles and ventral brushes on remarkably long and slender legs; and, above all, the special conformation of the gonopodal telopodite.

It is the latter that provides most of the characters useful for a confident separation of species in *Annamina* (see Key below). The gonotelopodite always shows an enlarged basal part of the femorite which is clearly flattened dorsoventrally. The femorite is set off from the acropodite by a distinct subtransverse sulcus or cingulum (**su**) that marks a postfemoral region more distally, a trait that, together with the presence of a number of femoral and postfemoral outgrowths or processes, allows the placement of the genus in Sulciferini (cf. Jeekel 1968). In *Annamina* spp., the femorite (**fe**) shows no longitudinal mesal groove so characteristic of numerous Sulciferini (*Oxidus* Cook, 1911, *Tylopus* Jeekel, 1968, *Sichotanus* Attems, 1914, *Cawjeekelia* Golovatch, 1980, etc.), but instead it has two more or less distinct lobes: one hyaline, rounded and ventral in position (**vl**), often folded mesad and especially hypertrophied in *A. attemsi* sp. n., the other a mesal, typically papillate or microdenticulate bulge (**ml**) which is particularly prominent in *A. xanthoptera* and *A. mikhaljovae* sp. n. The seminal groove in *Annamina*, unlike that in the remaining sulciferines, runs along the femorite not so much mesally as dorsally, to follow onto a surprisingly short and flagelliform solenomere (**sl**) detached on the dorsomesal face about the level of the postfemoral sulcus. This condition seems to be apomorphic, as is the complete absence of significant membranous elements in the solenophore which is represented by a rather small and short tooth (**t**), sometimes subtruncate, bi- or trifid, that subtends the distal part of the solenomere, leaving only the latter’s tip exposed beyond **t**. The solenophore tooth is located ventrally near the base (usually) or close to the midpoint (*A. attemsi* sp. n.) of a centro-apical process or spine (**a**), always hyaline, sometimes lobe-shaped, but more often acuminate, invariably the longest and largest of the postfemoral outgrowths. Process **a** can be flanked by two distinct, albeit shorter, processes: one lateral (**lp**), which is serrate in *A. xanthoptera* and *A. mikhaljovae* sp. n., but simple in *A. irinae* sp. n., the other mesal (**mp**) which is long in *A. mikhaljovae* sp. n., very short in *A. irinae* sp. n. and absent from the other two congeners. Process **lp** can also be rudimentary: *A. attemsi* sp. n.

Based on gonopodal structure alone, this latter species seems to be the most disjunct among congeners (see its diagnosis above and Key below). Since it seems to co-occur with *A. xanthoptera* near Danang, this observation agrees well with the general wisdom that two sympatric or even syntopic congeners tend to differ more strongly than others.

The above outline of the diversity of and variations in gonopodal characters in *Annamina* spp. helps us not only to better redefine the genus against the other Sulciferini, but also to key all of its four presently known species. Because the millipede fauna of Vietnam is the richest in Indochina, but still quite poorly known, it seems very likely that further *Annamina* species will be found in the future.

### Key to *Annamina* species (based on gonopodal characters)

**Table:** 

1	Postfemoral region of gonopod with a distinct, slender, slightly curved, mesal process (**mp**); apical process (**a**) broad and lobe-shaped (Fig. 11C–E)	***A. mikhaljovae* sp. n.**
–	Mesal process either very short or absent	**2**
2	Mesal process (**mp**) very short, mesal lobe (**ml**) of femorite ear-shaped (Fig. 9)	***A. irinae* sp. n.**
–	Mesal process absent	**3**
3	Femorite slender, ventral lobe (**vl**) hypertrophied, postfemoral lateral process (**lp**) rudimentary (Figs 6, 7)	***A. attemsi* sp. n.**
–	Femorite stout, ventral lobe (**vl**) small and inconspicuous, postfemoral lateral process (**lp**) very strong and serrate (Figs 2–4)	***A. xanthoptera***

## Supplementary Material

XML Treatment for Annamina

XML Treatment for Annamina xanthoptera

XML Treatment for Annamina attemsi

XML Treatment for Annamina irinae

XML Treatment for Annamina mikhaljovae
